# Specific interaction of zinc finger protein Com with RNA and the crystal structure of a self-complementary RNA duplex recognized by Com

**DOI:** 10.1371/journal.pone.0214481

**Published:** 2019-04-25

**Authors:** Martyna Nowacka, Humberto Fernandes, Agnieszka Kiliszek, Agata Bernat, Grzegorz Lach, Janusz M. Bujnicki

**Affiliations:** 1 International Institute of Molecular and Cell Biology in Warsaw, Warsaw, Poland; 2 Institute of Biochemistry and Biophysics, Polish Academy of Sciences, Warsaw, Poland; 3 Institute of Bioorganic Chemistry, Polish Academy of Sciences, Poznan, Poland; 4 Institute of Molecular Biology and Biotechnology, Faculty of Biology, Adam Mickiewicz University, Poznan, Poland; Erasmus Medical Center, NETHERLANDS

## Abstract

The bacteriophage Mu Com is a small zinc finger protein that binds to its cognate mom mRNA and activates its translation. The Mom protein, in turn, elicits a chemical modification (momification) of the bacteriophage genome, rendering the DNA resistant to cleavage by bacterial restriction endonucleases, and thereby protecting it from defense mechanisms of the host. We examined the basis of specificity in Com–RNA interactions by *in vitro* selection and probing of RNA structure. We demonstrated that Com recognizes a sequence motif within a hairpin-loop structure of its target RNA. Our data support the model of Com interaction with mom mRNA, in which Com binds to the short hairpin structure proximal to the so-called translation inhibition structure. We also observed that Com binds its target motif weakly if it is within an RNA duplex. These results suggest that the RNA structure, in addition to its sequence, is crucial for Com to recognize its target and that RNA conformational changes may constitute another level of Mom regulation. We determined a crystal structure of a Com binding site variant designed to form an RNA duplex preferentially. Our crystal model forms a 19-mer self-complementary double helix composed of the canonical and non-canonical base pairs. The helical parameters of crystalized RNA indicate why Com may bind it more weakly than a monomeric hairpin form.

## Introduction

RNA-binding proteins play important roles at every stage of RNA life cycle: transcription, splicing, editing, export, degradation and regulation of translation [1]. Many of them bind RNA molecules using RNA binding domains (RBDs), which exhibit a wide variety of structural forms as well as mechanisms of substrate recognition and binding. One of the most abundant RNA-binding domain types is a zinc-finger (ZnF) [2–4], which belongs to a large class of protein domains that stabilize their structures by tightly bound zinc ions [2]. Amongst them are classical ZnFs that were first discovered as DNA-binding domains [5]. Later on, it has been demonstrated that ZnFs also act as RNA or protein binders. ZnF-containing proteins can use just one ZnF domain to recognize and bind their substrate, e.g., GAGA-DBD [6] and SUP [7], or they can employ a whole array of ZnF domains, such as in TFIIIA [5] and WT1 [8]. ZnF domains that recognize and bind RNA substrates can do it in different ways. The binding usually involves hydrogen bonds, made by either ZnF’s backbone or side chain functional groups, and stacking interactions [2]. RNA binding by ZnFs can be either sequence-specific or non-sequence-specific, and in case of proteins containing ZnF arrays, a combination of both manners is possible. Sometimes the structure of the RNA substrate is important for binding; for instance ZRANB2s are single-stranded RNA-binding domains [9], JAZ preferentially binds to double-stranded RNA or RNA/DNA hybrids [10,11], whereas some ZnF modules of transcription factor TFIIIA recognize RNA bases that are in the ‘flipped-out’ conformations [2,12].

**Fig 1:**
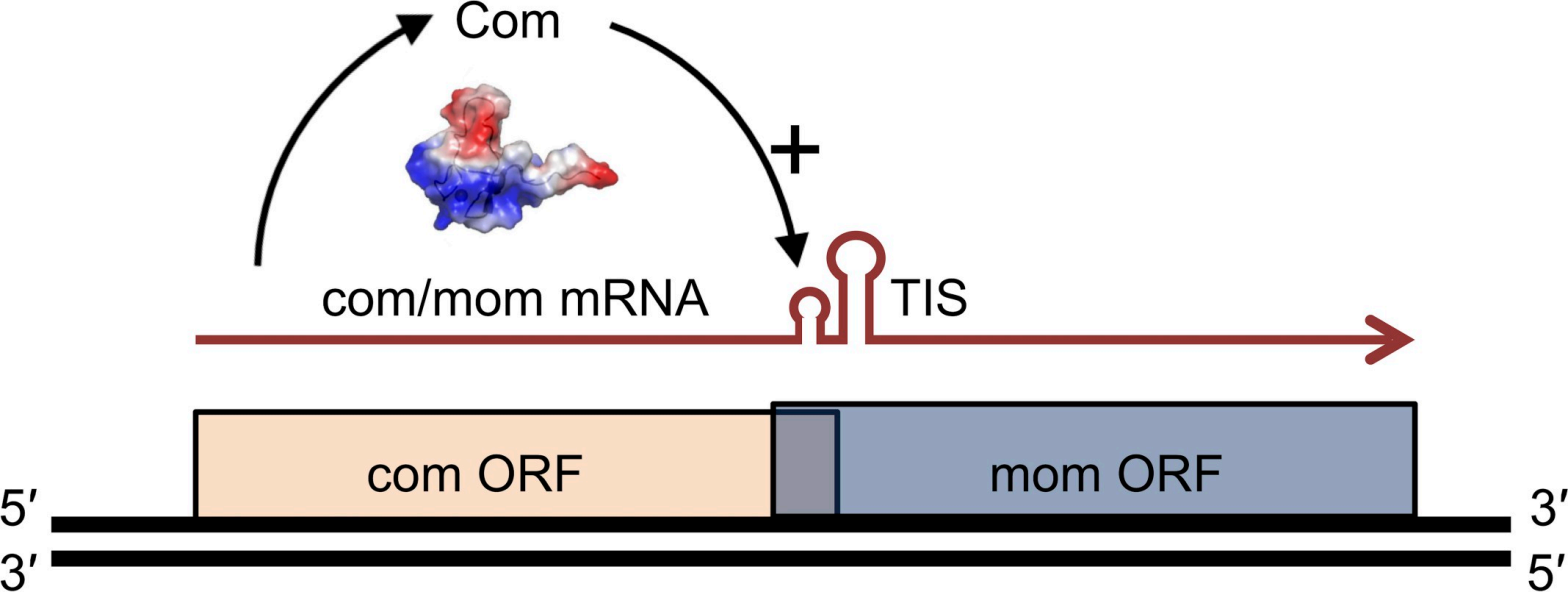
Schematic overview of bacteriophage com/mom operon regulation. Com binds to the hairpin (by the unknown mode) proximal to the TIS structure in the mom mRNA and promotes its translation.

The bacteriophage Mu Com-RNA complex can be considered one of the model systems for studying the interaction of a ZnF domain with RNA. The Com protein consists of 62 amino acids, including an N-terminal CCCC zinc finger module where four cysteine residues are involved in zinc ion coordination, and a C-terminal intrinsically disordered segment [13,14]. Com regulates the expression of the Mom system (Fig 1), responsible for chemical modification of the phage DNA and phage genome protection against a wide variety of bacterial restriction endonucleases [15]. So far, it has been proposed that Com targets the RNA hairpin-loop structure upstream to its cognate Mom mRNA translation start site, contributes to the changes in the mRNA secondary structure of the so-called translation inhibition structure (TIS) and, consequently, to the exposition of the translation start signals [15–17].

In this work, we aimed at establishing the preferred RNA sequence recognized by Com and determining the structural basis of Com-RNA interactions. We attempted to co-crystallize Com with its natural RNA target, as well as with its variants designed computationally to form either a monomeric hairpin-loop structure or a homo-duplex form. Thus far, we obtained crystals and solved the structure of an RNA homo-duplex form. While the high-resolution structure of the Com-RNA complex remains to be determined, we propose a low-resolution structural model of Com-RNA interactions based on the available experimental data.

## Materials and methods

### Cloning, expression, and purification of Com

The *Haemophilus sputorum* Mu-like prophage Com gene sequence (GI:400376712) was optimized for *Escherichia coli* expression and synthesized by GeneArt Gene Synthesis (Thermo Fisher Scientific). The gene was subcloned into prokaryotic expression pGEX4T vector (GE Healthcare Life Sciences). *E*. *coli* BL21(DE3) (New England Biolabs) was used to overexpressed the glutathione S-transferase (GST) fusion-tagged Com that included a two-residue linker (S-H) between the GST tag and Com. Expression was carried out in LB medium, induced with 1 mM isopropyl-D-1-thiogalactopyranoside solution (IPTG) at OD_600_ of 0.6 and conducted at 37°C with shaking at 200 rpm for 4 hours. The LB medium was supplemented with 100 μM ZnSO_4_ (Sigma-Aldrich) upon induction. The fusion protein was purified by GST affinity with Glutathione-Agarose beads (Sigma-Aldrich) according to the manufacturer protocol and stored in +4°C.

### SELEX

The Systematic Evolution of Ligands by Exponential enrichment (SELEX) was used to determine the specificity of Com binding with RNA. SELEX was carried out, as described previously by Skrisovska *et al*. [18] and Cavaloc *et al*. [19]. The starting matrix of DNA oligonucleotides was as follows: 5ʹ GCGTCTCTGCAGTAGTTA(N20)AGTCGGCATCTTGGTACCCTATAGTGAGTCGTATTACC3ʹ (where N20 indicates a 20 bases random sequence), and 5'GGTAATACGACTCACTATAGGGTACCAAGATGCCGACT3ʹ (Metabion). After the fifth cycle of selection, the RT-PCR products were subjected to Next-Generation Sequencing on MiSeq (Illumina) platform (Oligo.pl, Warsaw, Poland).

The RNA sequence consensus motif was generated by the motif discovery tool MEME (Multiple Em for Motif Elicitation) [20] with default parameters and width of the motif set for maximum seven nucleotide residues. The gapped RNA motif comprising two repeats of the binding site was generated by the GLAM2 (Gapped Local Alignment of Motifs 2) method [21] and was defined as NGAGNNCC(N)_2-3_GAGNNCCNN, where N refers to any nucleotide residue.

### RNA design

The sequence of the native Com target folds preferentially into a hairpin but can dimerize to form a largely helical duplex, depending on conditions [22]. Based on the RNA sequence motif obtained from SELEX, RNA molecules preferentially folding either into the monomeric or the dimeric form were computationally designed using DesiRNA (G.L. and J.M.B., unpublished, software available for download at http://iimcb.genesilico.pl/desirna/, and at https://github.com/GrzegorzLach/DesiRNA). The optimized parameter was the difference between the free energies of the dimer and of the separated strands folded into monomeric hairpin-loop structures. This difference is directly related to the equilibrium constant of the dimer formation and has been either minimized or maximized, to produce sequences containing the conserved motif that exhibit strong propensity to form either a dimer or a monomer. The free energies have been computed using the McCaskill algorithm [23] and Turner parameters implemented in the ViennaRNA package RNAlib library (version 2.1.2) [24].

Sequences of designed monomeric RNAs were as follows: RNA I 5'CGAGAACCAGAGAGUUCCGG3', RNA IA 5'CUGCAACCAGAGAGUUGCGG3', RNA IB 5'CGCGUACAAGUGAGUACCGG3'. The sequence of designed dimeric RNAII was 5'AGAGAACCCGGAGUUCCCU3'. The native 19-mer Mom RNA sequence was: 5'GAAUGCCUGCGAGCAUCCC3'. All the oligonucleotides were chemically synthesized by FutureSynthesis, Poznan, Poland.

### EMSA

The RNA-protein interactions were determined by the Electrophoretic Mobility Shift Assay (EMSA). We used the affinity purified GST-fused Com and [^32^P] labeled synthetic RNAs. RNAs were labeled with γ ^33^P ATP (Hartmann Analytic) using T4 PNK Kinase (Thermo Fisher Scientific), phenol/chloroform extracted, precipitated, desalted and separated from the unincorporated label on MicroSpin G-25 Columns (GE Healthcare Life Sciences). RNA samples were annealed in buffer containing 50 mM Tris, pH 7, 2.5 mM MgCl_2_ by heating at 80°C for 15 min and cooling 1°C per min until it reached room temperature (performed in Thermal Cycler BioRad).

Each binding reaction consisted of about 4 pmole of annealed RNA and appropriate Com concentration: 0, 8, 16, 32, 64, 125, 250, 500, 1000, 2000 nM (the RNA IA and IB motif tests were performed in the presence of 2000 nM Com). The binding reaction was carried out for 1 hour at room temperature in the reaction buffer containing: 50 mM Tris, pH 7, 50 mM NaCl, 1 μg of BSA, 1 mM DTT, 10% glycerol, 0.1% Igepal, 2.5 mM MgCl_2_, 20 μM ZnSO_4_, 2 μg dI-dC. The samples were then loaded on a 15% non-denaturing polyacrylamide gel and resolved in 0.5x Tris-borate-EDTA (TBE) buffer at 1 W for 1.5 hours. The separated RNA samples were visualized using Typhoon Phosphorimager.

### Probing of RNA structure by SHAPE

The chemical probing of RNA molecules using the SHAPE method was carried out in triplicates, as described previously by Wilkinson *et al*. [25]. The genetic constructs contained DNA sequences for RNA I, RNA IA, RNA II, 19-mer Mom RNA (described in the RNA designing subchapter) embedded within a SHAPE cassette that contained 5ʹ and 3ʹ flanking sequences covering a unique primer binding sites. The SHAPE cassette did not interfere with the folding of internal RNA. The constructs were synthesized by GeneArt Gene Synthesis (Thermo Fisher Scientific). The RNAs were transcribed *in vitro* using the FlashScribe kit (Invitrogen), according to the manufacturer’s protocol.

The SHAPE probing reactions were carried out for each RNA alone and for RNAs crosslinked with the Com protein. For footprinting of Com binding sites, an excess of Com was added to RNA after the annealing step (250 pmole of Com protein per 2 pmole of RNA) and the mixed samples were incubated for 30 min at room temperature. Next, the Com protein was crosslinked to RNA with a 254 nm wavelength light in the Ultraviolet Crosslinker (UVP, LLC) for 30 min. During crosslinking the samples were kept on ice, 15 cm from the light source. At the same time, the annealed RNA for RNA probing reactions (without the Com protein) was kept at room temperature for one hour. The probing of all samples was carried out with 9 mM NMIA (N-methylisatoic anhydride) (Thermo Fisher Scientific) for 30 min. DMSO was used in control reactions. RNA was reverse-transcribed with SuperScript III (Invitrogen), in the presence of fluorescent-labeled primers (VIC and NED, Thermo Fisher Scientific). The DNA obtained was capillary sequenced (Oligo.pl, Warsaw, Poland) and the SHAPE results were analyzed with the qshape software [26].

### RNA structure prediction and visualization

RNA secondary structure was predicted with RNAstructure [27], using reactivity from the SHAPE experiments as pseudo-free-energy constraints. Secondary structure was visualized by VARNA [28].

### Crystallization and X-ray data collection

Chemically synthesized oligoribonucleotides: RNA I, RNA II and 19-mer Mom RNA, 60 nmol (80 μg), were dissolved in 5 μl of buffer containing: 50 mM HEPES pH 7, 100 mM NaCl, 2.5 mM MgCl_2_ and annealed by heating at 80°C for 15 min and cooling 1°C per min until the solution reached room temperature. RNA was added to 100 μl of affinity-purified GST-Com fusion protein solution concentrated to 8 mg/ml in the crystallization buffer containing 50 mM HEPES, pH 7, 100 mM NaCl, 1 mM DTT. The Phoenix nano-dispensing robot was used to set 0.2 μl crystallization drops with the Index Screen and Crystal Screen (Hampton Research) in 96 well crystallization plates (Hampton Research). It took 2–4 days for first rocky crystals to appear in Crystal Screen D11 condition. No further optimization from initial screening was carried out. Crystals were cryoprotected for 10 sec. in reservoir solution supplemented with 20% PEG400, flash-frozen, and stored in liquid nitrogen. The crystal collected from the Crystal Screen D11 crystallization condition (0.1 M sodium acetate trihydrate pH 4.6, 2M ammonium sulfate) was used for X-ray data collection at Bessy synchrotron 14.2 beamline (Berlin, Germany) (Gerlach, Mueller & Weiss, 2016). The data were indexed and scaled by XDS [29] to 2.27 Å resolution.

### Structure determination and refinement

An estimation of the number of molecules in the asymmetric unit [30] indicated the presence of a 19-mer RNA duplex with a solvent content of 56.1%. The search model used for molecular replacement was a crystal structure of an 8-mer RNA duplex, PDB code 3GLP [31]. Phases were determined using Phaser [32]. The rotation/translation search led to a Z score of 8.1 and a final log-likelihood gain (LLG) of 350. The solution consisted of two sequentially stacked 8-mer search models, with a single base pair gap between them. The initial model was improved by several rounds of model building using Coot [33] and refinement. Initial refinement was carried out using Refmac5 [34] from the CCP4 program suite [35] and then continued with PHENIX [36]. Solvent molecules were manually modeled using Coot after the RNA duplex was fully built and refined. Intramolecular interactions, including canonical and non-canonical base pairs and stacking interactions, were analyzed by ClaRNA [37]. Atomic coordinates of the crystallographic model were deposited in the Protein Data Bank (accession code 6IA2).

## Results

### Identification of Com RNA targets by SELEX

**Fig 2:**
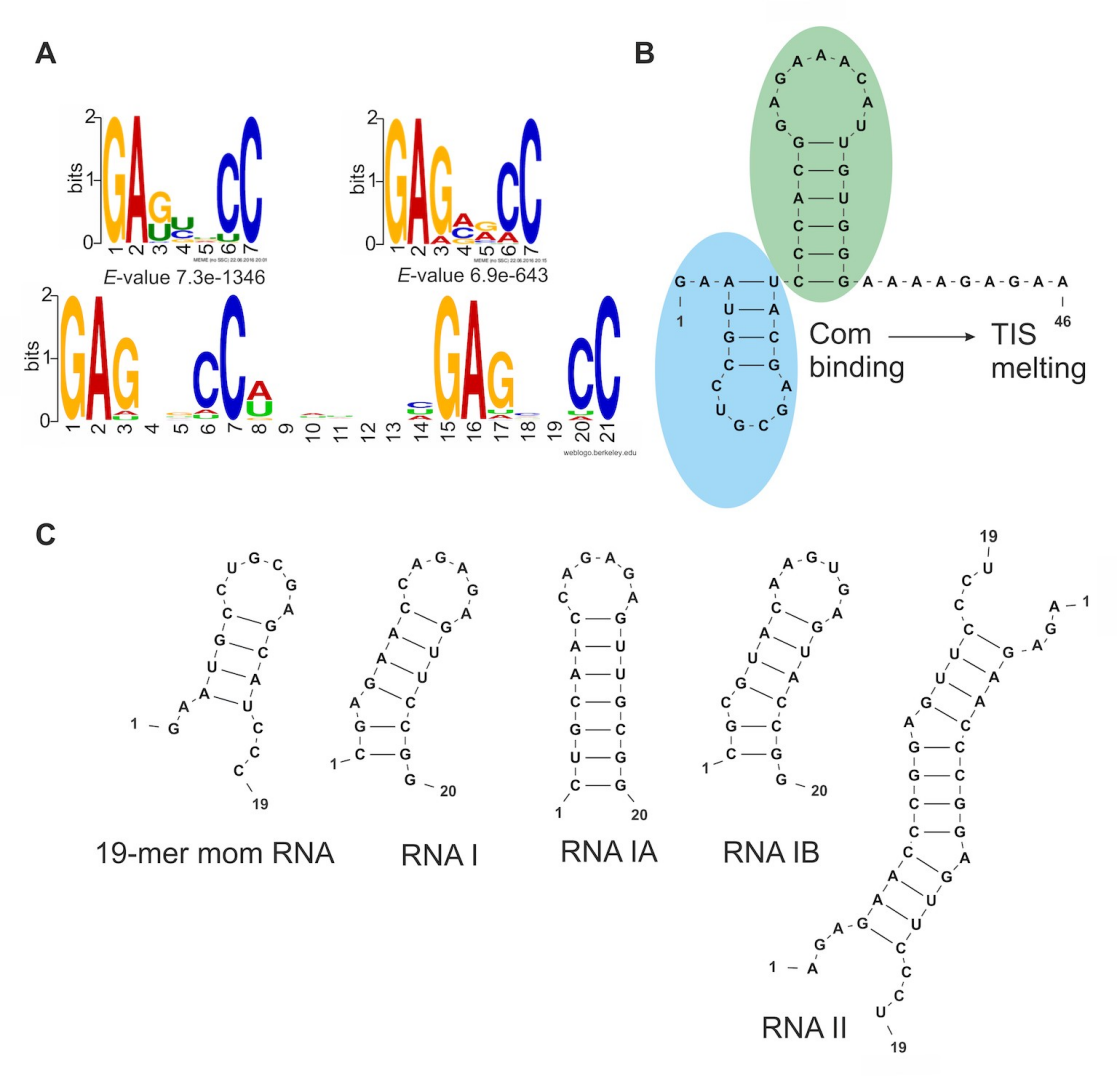
RNA molecules and sequence motifs presented in the studies. (A) Consensus RNA sequence motifs obtained after SELEX. Two single motifs were generated by MEME [20]. The bipartite motif was created by GLAM2 [21] and WebLogo [39]. (B) Mom regulatory region containing TIS structure (in green) and Com binding region– 19-mer Mom RNA fragment (in blue). (C) RNA molecules used in the presented studies. Secondary structure was predicted with RNAstructure Web Server.

In order to characterize the RNA substrate preference of the Com protein, we carried out an *in vitro* selection experiment using the SELEX method (see Methods for details). As a protein bait, we used the zinc finger domain of the *H*. *sputorum* Com. After five cycles of SELEX, we found two very similar RNA motifs, both with the core sequence 5ʹ-GAG(N)_2_CC-3ʹ, where N refers to any nucleotide (Fig 2A) (first was present in all and second in 637 per 999 sequences analyzed). The GLAM2 method, which is able to perform gapped motif discovery showed that the majority of selected RNA sequences had a bipartite motif with two instances of the 5ʹ-GAG(N)_2_CC-3ʹ sequence (Fig 2A) (present in 868 per 999 sequences analyzed). Secondary structure predictions indicated that the two core sequences were symmetrically localized in loops at both sides of a 3–4 nucleotide long stem. The stem consisted of variable sequence, but the pair closing the loop was invariably C-G.

### Com binding to selected RNA is sequence- and structure-specific

**Fig 3:**
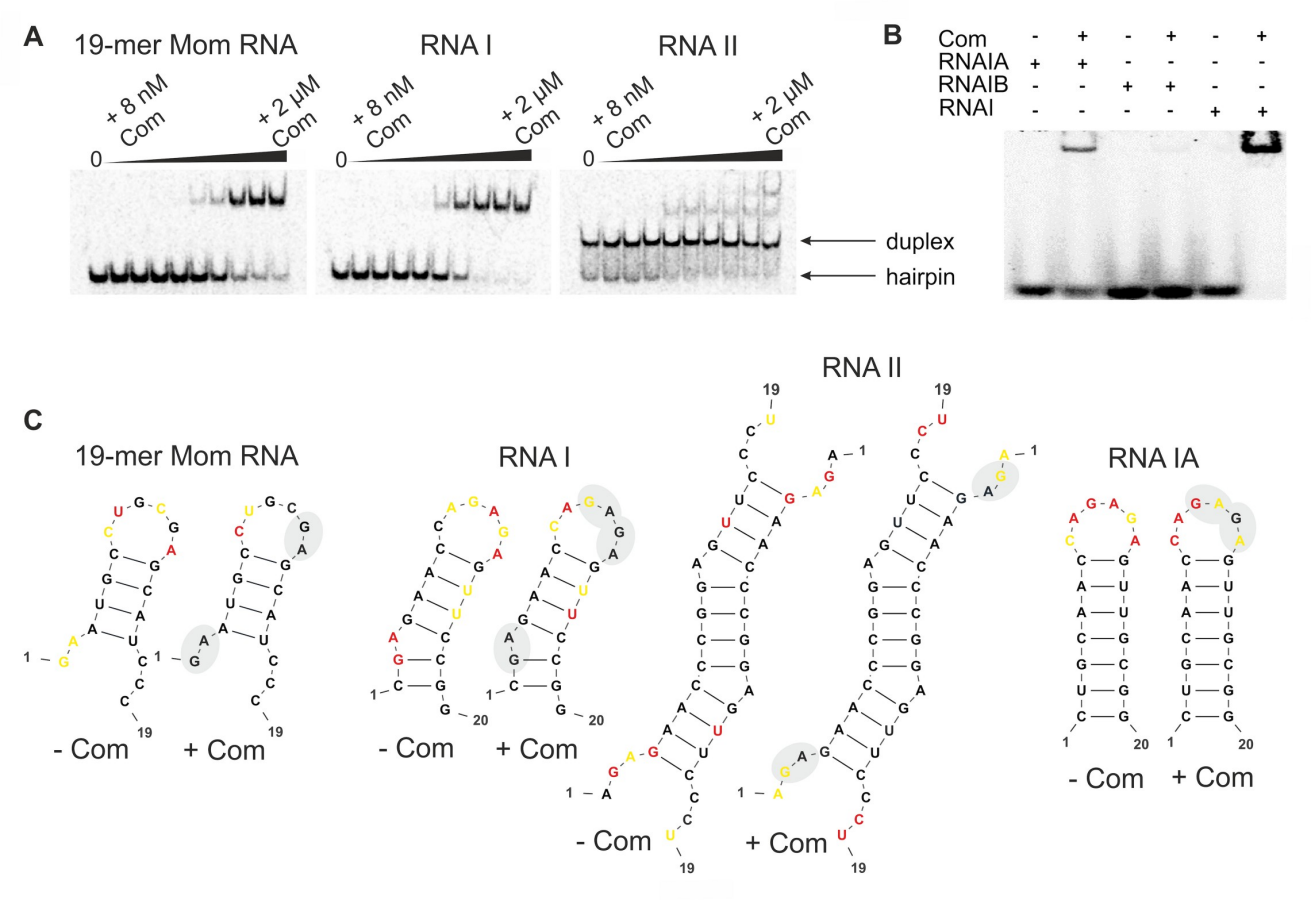
Binding assays and secondary structure models for RNA molecules containing the Com binding sites. (A) Electrophoretic mobility shift assays confirmed that the Com is able to bind 19-mer Mom RNA (a fragment of the regulatory region of Mom mRNA) as well as RNA I and RNA II. (B) EMSA confirmed that the Com binds RNA with 5ʹ-CC(N)_2-3_GAG-3ʹ motif. (C) Secondary structures of Com targets predicted by RNA structure [27] using reactivity from the SHAPE experiment as pseudo-free-energy constraints for RNA molecule alone (first molecule of each pair) and for the same RNA in the presence of Com (second molecule of each pair). Residue symbols are color-coded according to SHAPE reactivity: red—high reactivity (≥ 0.85), orange–moderate reactivity (≥ 0.4, < 0.85), black–weak reactivity (< 0.4). The GA residues with decreased reactivity upon Com binding are indicated with the gray shade.

RNA sequences found during *in vitro* selection were predicted to form a hairpin. However, RNA hairpins at high concentration could also form a self-complementary duplex [22,38]. We decided to examine which of the two possible RNA structures (a monomeric stem-loop or a dimeric stem) was preferred by Com. Therefore, we designed two RNA molecules folding preferentially into either of the forms and tested them for Com binding. As a control, we used the 19-mer Mom RNA fragment that preferably formed the hairpin form [22] (Fig 2B and 2C). One designed molecule, called RNA I, was expected to fold into a hairpin structure at 1 mM concentration (Fig 2C). The other molecule, RNA II, at the same concentration was expected to form a self-complementary duplex (Fig 2C). All RNAs contained the consensus motif recognized by Com. The monomeric form of RNA I, 19-mer Mom RNA as well as a dimeric form of RNA II were confirmed by non-denaturing PAGE (with the slower migration of the RNA duplex band) (Fig 3A). As seen on the gels after EMSA, some RNA II molecules also acquired a monomeric, hairpin form (at the same time and under the same conditions, we did not notice a dimeric form of RNA I and 19-mer Mom RNA).

We observed binding of Com to both designed RNAs, as well as to the 19-mer Mom RNA control, indicated by shifted bands on the gel after EMSA (Fig 3A). We observed that Com preferentially bound RNA hairpins (i.e., the monomeric version of the RNA substrate). The self-complementary duplex was targeted only after all RNA hairpin molecules were bound, as demonstrated by a super-shift in mobility of the RNA duplex (Fig 3A).

To establish the RNA-Com binding site, we checked which variant of the single RNA motif within the hairpin bipartite motif of RNA I (Fig 2C) is actually bound by Com. To this end we modified the sequence of RNA I (while preserving its hairpin structure) in order to eliminate one or the other part of the bipartite RNA motif: the RNA IA contained only 5ʹ-CC(N)_2-3_GAG-3ʹ present in the loop, and the RNA IB contained only 5ʹ-GAG(N)_2_CC-3ʹ present in the 3ʹ half of the stem-loop RNA (Fig 2C). We observed binding of Com to the RNA IA and very little binding to the RNA IB (Fig 3B). However, the binding of Com protein to the RNA IA (with just one motif) was less efficient than its binding to the RNA I (with two motifs).

In the next step, we decided to probe the binding of Com to it RNA targets (19-mer Mom RNA, RNA I, RNA IA, and RNA II; RNA IB was excluded due to lack of binding to Com (Fig 3B)). To examine the secondary structure of Com binding sites, we carried out structure probing by SHAPE. First, we probed each RNA alone and then we used the SHAPE method to perform RNA footprinting in complex with Com. The secondary structure models of RNAs obtained on the basis of our SHAPE (Fig 3C) was in agreement with our *in silico* predictions and the earlier models proposed by Hattman *et al*. [13] and Wulczyn & Kahmann [16]. The 19-mer Mom RNA, RNA I and RNA IA were predicted to form a short RNA hairpin with loops of six residues and the RNA II was predicted to form a duplex with unpaired ends and wobble adenines. In the earlier models of Mom regulatory region, the predicted Com binding site was located in the loop of a short hairpin proximal to the TIS (also in the hairpin form, Fig 2B), which included the Mom GUG start codon in its stem. The footprinting results indicated that the GA dinucleotide of the consensus sequence motif was somehow involved in Com binding, as indicated by a substantial decrease in SHAPE reactivity in the examined Com-RNAs crosslinked samples, in comparison to the free RNA samples (Fig 3C). We also notice a decrease in reactivity of the residue proceeding GA for all Com-RNA-cross-linked samples as well as of cytosine (the first nucleotide of the loop) for all RNAs in the hairpin form.

In earlier chemical and enzymatic footprinting studies of Hattman *et al*. [13] and Wulczyn & Kahmann [16], the A residue of the GA dinucleotide was consistently predicted as important for Com binding. However, the G residue in the presence of Com was indicated as sensitive to cleavage by T1 RNase. Since this RNase recognizes only unpaired G residues, the G involvement in Com binging was inconclusive [16]. SHAPE and RNase T1 probe different structural features: SHAPE indicates flexible residues whereas T1 cleaves phosphodiester bond after single-stranded guanosines. Thus, G residue although being single-stranded could be more rigid in the presence of Com.

In earlier studies, the CC dinucleotides, both, proceeding and following the SHAPE-reactive GA, were sensitive to double-strand-specific CV1 nuclease cleavage, but only in the absence of Com and they were unreactive or weakly reactive to chemical probes, regardless of the absence or presence of Com [15,16]. This suggested its double-stranded form and Com proximity during binding. In our studies, only the first C of the CC dinucleotide remained weakly reactive in the hairpin RNAs upon Com binding, suggesting that it was rigid and most probably base-paired; however, the lack of reactivity due to Com proximity cannot be excluded. The second C of the CC dinucleotide could actually interact with G of GA dinucleotide in the loop, gaining some properties of a paired nucleotide in the free RNA form. However, during the engagement of GA in Com binding, the C in the loop became more susceptible to SHAPE reagent.

The 19-mer Mom RNA, RNA I, and RNA II analyzed by SHAPE had more than one GA dinucleotide in the context of the bipartite consensus sequence motif obtained in our SELEX experiment. Interestingly, we noticed a decrease in SHAPE reactivity for both GA dinucleotide in the examined Com-19-mer Mom RNA, Com-RNA I and Com-RNA II crosslinked samples (Fig 3C). This may indicate that more than one Com binding site is present and utilized in the Mom regulatory region, or/and that the RNA structure rearrangements triggered by Com binding in one position, expose other binding sites which are consequently occupied by Com in the next step of Mom regulation. Alternatively, the occupancy of Com in another GA motif, shown in our SHAPE experiment may be explained by dimerization of GST tag of the GST-Com fusion protein used in the study or by a high excess of protein (125:1 molar ratio) used for crosslinking with RNA.

### Crystal structure of RNA II duplex

**Fig 4:**
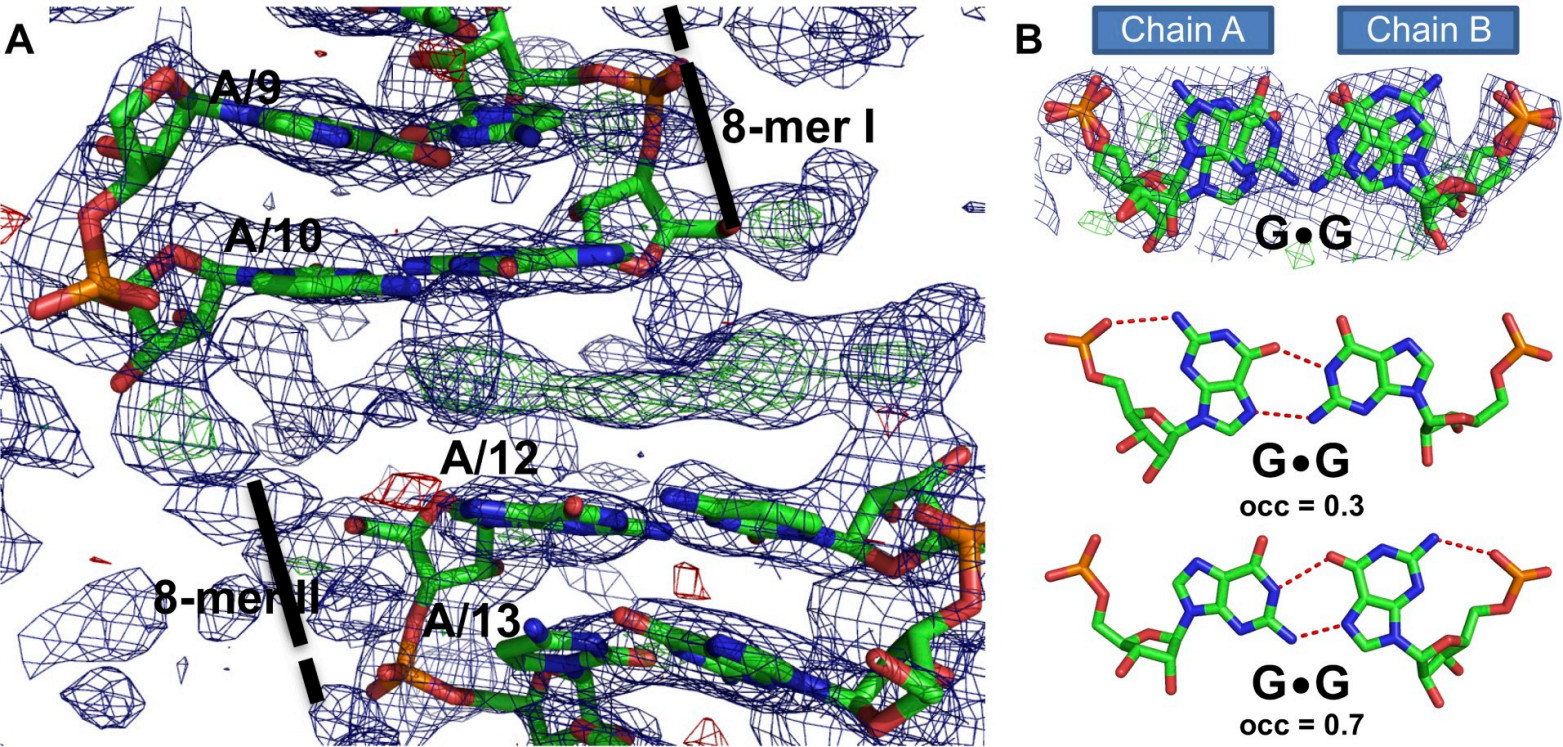
Overview of RNA II structure. (A) Molecular replacement solution with two 8-mer molecules in stick representation, and respective electron density (2Fo-Fc map) contoured at 1.0σ level–blue mesh. The FoFc maps contoured at the 3.0σ level are shown in green and red meshes. Nucleotide labels correspond to chain A of RNA II. (B) The overview of non-canonical G•G base pair at position 10 presents the overlap of the double conformation with electron density and G•G pair with occupancy 0.3, and 0.7. The hydrogen bonds between the bases are shown as red dash lines. The figure was prepared in PyMol [43].

To better understand interactions of ZnF Com with its RNA target, we attempted to co-crystallize the GST-Com protein with the hairpins: RNA I and the 19-mer Mom RNA fragment, as well as dimeric RNA II. We were able to obtain crystals and to collect and process X-ray data only for samples where GST-Com was supplemented with RNA II. Data collection statistics are summarized in Table 1. The initial estimation of asymmetric unit content revealed, however, that the packing of a putative GST-Com:RNA II complex was unfeasible, meaning that the macromolecule(s) crystallized were of smaller size. According to the computational analysis structure factors with the RIBER/DIBER server [40], the crystal contained only RNA and no protein with 94% probability. The presence of an RNA homo-duplex in the crystal was then definitely proven by the molecular replacement solution using Phaser [32]. The top solution from molecular replacement had good statistics, and electron density maps with good quality. The maps also corresponded to the first two nucleotides at the 5ʹ end of the RNA II sequence (not present in the search model), and a clearly visible gap between the 8-mer RNA model duplexes (Fig 4A). Thus the molecular replacement solution provided continues density for the 19-mer homo-duplex of the RNA II molecule.

**Table 1:** Data collection and structure-refinement statistics.

Data collection	
Diffraction source	Bessy 14.2
Rotation range per image (°)	1
Total rotation range (°)	180
Crystal-to-detector distance (mm)	270
Space group	*P* 2_1_ 2_1_ 2_1_
Unit-cell parameters (Å)	31.8 38.8 101.9
Mosaicity (°)	0.3
Resolution range (Å)	31.80–2.27 (2.41–2.27)
Total No. of reflections	42351
No. of unique reflections	6200
Completeness (%)	99.4 (98.6)
Multiplicity	6.8 (6.14)
<I/σ(I)> ^a^	27.1 (3.5)
*R*_meas_ (%) ^b^	5.0 (54.9)
Refinement	
Program	Phenix
Overall mean B-factor (Å^2^) (chain A; B; C; D; E)	40.1; 42.2, 54.3; 68.2; 40.1
*R*_work_ (%) ^c^	21.19
*R*_free_ (%) ^d^	24.60
RMSD of bonds (Å)	0.005
RMSD of angles (°)	0.911

Values in parenthesis are for the outer shell.

^a^ <*I*/σ(*I*)> is the mean signal-to-noise ratio, where *I* is the integrated intensity of a measured reflection and σ(*I*) is the estimated error in the measurement.

^b^ *R*_meas_ = 100 × Σ*hkl*{*N*(*hkl*)/[*N*(*hkl*)-1]}^1/2^Σ_*i*_|*I*_*i*_(*hkl*)—<*I*(*hkl*)˃|/ Σ*hkl* Σ_*i*_ *I*_*i*_ (*hkl*) where *Ii*(*hkl*) and ⟨*Ii*(*hkl*)⟩ are the intensity of measurement *i* and the mean intensity for the reflection with indices hkl, respectively.

c *R*work = 100 × Σ*hkl* || *F*obs| − |*F*calc || /Σ*hkl*|*F*obs|, where *F*obs and *F*calc are observed and calculated structure-factor amplitudes, respectively.

^d^ *R*free is the *R*work calculated using a randomly selected 5% sample of reflection data that were omitted from the refinement.

The initial *R* factors (40.98/43.10 for *R* factor and *R*free, respectively) were improved to the final 21.19/24.60, by the addition of the missing nucleotides, sequence correction, introduction of a double conformation for the central G•G pair (and consequently also for G at position 11 in chain B), and the addition of solvent molecules.

The 19-mer self-complementary RNA II duplex folds into a double helix of the A-form (A-RNA). In the crystal lattice, along with the two-one screw axis parallel to c, the duplexes stack end to end forming a pseudo-continuous helix (S1 Fig). The RNA duplex consists of 14 canonical Watson-Crick base pairs (A-U, C-G) and 5 non-canonical base pairs (four A•C and one G•G pair in the middle of the duplex (Fig 4B)). All non-canonical base pairs form two hydrogen bonds.

The central G•G base pair, although showing static disorder, breaks the chemical and crystallographic symmetry of the helix. This non-canonical pair was modeled as two alternative conformations: one with syn-anti and other with the anti-syn orientation of base rings. These two possibilities were modeled with 0.7 and 0.3 occupancies, respectively. Both guanine rings are flipped in respect to one another with an ~180 degree rotation around the C1’-N9 bond (no flipping is observed for the next residue–G at position 11 in chain B, which is also present in a double conformation). The interacting guanosine residues can be described as a Watson-Crick–Hoogsteen cis pair, according to ClaRNA classifier [37]. The two hydrogen bonds are formed between the N1-O6 and N2-N7 atoms of the guanines (the distance vary between 2.5 and 3.0 Å). An additional hydrogen bond is formed between the exo-amino group of G(syn) residues and its phosphate oxygen atom (2.86–3.23 Å), which further stabilizes the conformation of the central G•G pair.

All the other base pairs in RNA II show canonical Watson-Crick cis conformations. The distances between the C1' atoms of the paired residues are clearly different for the non-canonical G•G pair (S2 Fig). The C1’ atoms of the guanosine residues are separated by 11.0 or 11.6 Å, which is ~0.5 or 1.1 Å longer than for all canonical Watson-Crick pairs (average distance is 10.5 Å). In the case of A•C the C1ʹ-C1ʹ distances are slightly shorter (average is 10.2 Å). The average rise parameter calculated between each neighboring residues is 2.8 Å with a standard deviation of 0.35 Å (helical rise was calculated in W3DNA by the projection of the vector connecting consecutive C1’-C1’ middle points onto the helix axis [41]). The largest raise value (3.4 Å) is observed between the 12A and 13G residues in chain A while the minimum (2.2 Å) between 1A and 2G of chain A (S2 Fig). The presence of non-canonical base pairs, with the loosening of the helix packing, results in greater local tilt, roll, and twist of the G•G base pair and the neighboring bases. In respect to the λ angles, measured between the N-glycosidic bond and C1'-C1' atoms of paired residues, they range within ~50–60° for the typical Watson-Crick base pairs, whereas in the G•G pair they are between 25 and 66°. Of notice, also the non-canonical A•C pairs show a decrease of λ angles of ~5° for adenine bases and an increase of ~10° for the cytosine bases, independently of the chain (S2 Fig). The changes in values of helical twist are also associated with the non-canonical base pairs. For AG/CC steps, the helical twists is 23.5 and 28.5° while for GA/CC steps are 34.9 and 37.7°. In the case of GG/CG step the helical twist shows the lowest value– 22.3°. Unwinding and twisting are observed locally resulting in an average helical twist of 31° typical for A-RNA.

The solvent molecules in the crystal comprise a Cl- anion, a sulfate ion, and 10 water molecules. The sulfate ion was modeled at 0.7 occupancy, and all the other solvent molecules were modeled at full occupancy. The sulfate anion is found in the major groove, interacting with G10 of chain B, namely with the Watson-Crick edge of G(syn), as observed previously in similar circumstances [42]. The Cl- anion is located on the opposite side of the RNA helix, minor groove, and bound to G11 of chain A.

In general, the presence of non-canonical base pairs G•G and C•A does not distort the A-RNA form of RNA II, and the overall structure is stable, with the characteristic 11 base pairs per turn, the C3' endo conformation of the sugar rings, and the presence of the axial hole. The syn-anti arrangement of the central G•G pair allows minimizing the effect of guanines bulkiness and avoiding the steric clash between them. The non-canonical A•C pairs have G•U wobble-like conformation. The occurrence of double hydrogen bonding for the A•C pair is likely due to protonation of the adenine, on nitrogen in position 1, which can be promoted in the acidic crystallization condition (pH 4.6), but we cannot rule out that one of the bases is present as an imino tautomer, thus allowing two hydrogen bonds to be formed. Thus, weaker interactions are expected at higher pH, offering an explanation for the fact that some of the RNA II molecules were observed in the monomeric form in our EMSA experiment.

## Discussion

Com is a small zinc finger protein that regulates the translation of bacteriophage Mu Mom RNA. The regulation occurs via Mom RNA binding and structural rearrangements leading to an exposition of translation start site. Here we demonstrated that Com zinc finger is sufficient for RNA binding in sequence and structure-specific manner.

### Overview of Com interaction with RNA

Taking into account our data obtained by SELEX and SHAPE probing, we propose that the Com protein recognizes the 5ʹ-CC(N)_2-3_GAG-3ʹ motif present in the loop structure of an RNA hairpin that can be formed by its physiological target. The accessible GA dinucleotide is crucial for this recognition.

Multiple sequence motifs, similar to our bipartite SELEX-derived motif, are also present in the bacteriophage Mu Mom translation initiation region, which was already predicted [15], and confirmed in this study, to be a Com target. The motifs are localized in the region proximal to the TIS structure, which partially overlays TIS (5' **GAA**UG**CC**UGC**GAG**CAU**CC**CACG**GAG** 3'). Moreover, our results indicated that the Com recognition and binding of RNA required not only defined RNA sequence motif, but most probably the motif has to be positioned within a specific secondary structure. The results obtained herein support the model of Mom regulation by Com and definition of the Com target structure determined earlier by enzymatic and chemical probing [15]. In this respect, Com specifically binds the putative RNA target region, presumably contained in the stem-loop, and its binding destabilizes and melts the TIS structure [15]. We did not observe the transition stage of hairpin structure melting after Com binding in our SHAPE footprinting experiment. The explanation for this can be the lack of the C-terminal intrinsically disordered segment in the truncated version of Com used in our studies. This part of Com is dispensable for RNA binding (Fig 3A and 3B). However, it may be necessary for further structural RNA rearrangement and regulation of Mom.

Lima and coworkers presented contradictory results of Com binding assays. In their EMSA experiments, Com preferentially bound a 19-mer self-complementary RNA duplex (observed at about 1.55 mM concentration) and did not generate a mobility shift for the RNA hairpin form [22]. Although it is difficult to reconcile the lack of hairpin binding in studies reported by Lima *et al*., the strong binding of the RNA duplex might be explained by the differences in the structures of both duplexes, the 19-mer Mom RNA fragment [22] and 19-mer RNA II, used in our studies.

### Comparison of RNA II and Mom RNA duplex structures

**Fig 5:**
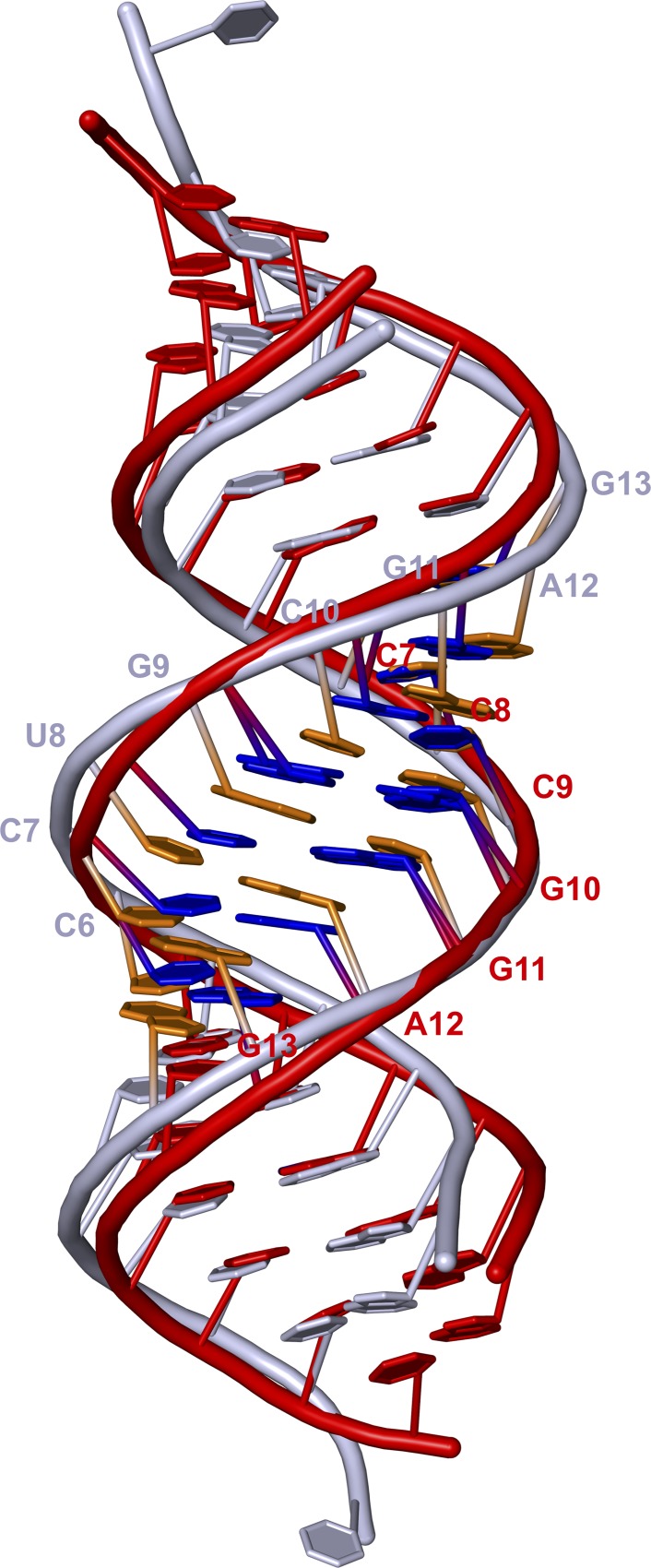
Superposition of the RNA II and 1KFO structures. RNA II (red) duplex structure with one CC(N)_2_GAG motif marked (bases in blue). 1KFO (gray) crystallographic duplex with one CC(N)_3_GAG motif marked (bases in orange).

The helical structure of the RNA II presented in this work is similar to the structure of 19-mer Mom RNA fragment published by Lima and colleagues [22]. Nonetheless, the stacking of bases in our RNA II homo-duplex is slightly looser. The biggest rise for the Mom RNA structure (PDB access number 1KFO) is 3.1 Å that is 0.3 Å smaller than the maximum rise measurement observed for our RNA II (although, the average rise for the Mom RNA structure is 2.7 Å, which is only 0.1 Å smaller than the average rise observed in our study). In general, the distance from the more distanced phosphates is 49.9–50.7 Å (chain A and B, respectively) for RNA II, and 48.3 Å in the 19-mer Mom RNA fragment. Both duplexes superimpose with a rmsd of 2.07 Å when all atoms are used, and 1.85 Å when only the backbone atoms are used. The 19-mer Mom RNA duplex analyzed earlier has a single nucleotide 3ʹ overhang, while RNA II has blunt ends, which explains why the biggest differences are observed at both ends of compared structures (Fig 5). Both, RNA II and a 19-mer Mom RNA duplex, contain the internal sequence motif recognized by Com, however, with different distances between CC and GAG sequences (2 and 3 nucleotides, respectively) as well as the GA dinucleotide at the beginning of each strand. Both of them have the A•C non-canonical base pair in the context of the internal GA dinucleotides, crucial for recognition by Com. An A•C base pair has been already speculated by Lima *et al*. to be able to introduce structural flexibility due to adenine N1 protonation and to function as a key element of a conformational switch, which can be triggered by environmental factors [22].

The internal GA dinucleotide of 19-mer Mom RNA duplex crystalized by Lima and colleagues [22] is involved in tandem wobble pairs A•C/G•U. This could potentially make this element even more suitable for weakening the stability of the RNA helix and making it prone to conformational rearrangements. In contrast, RNA II contains a G•G base pair, which is stabilized by three hydrogen bonds (including one internal hydrogen bond), adjacent to the internal GA dinucleotide. In RNA II, the G residue of the GA dinucleotide is involved in canonical base pairing (S1 Fig). This situation may reduce the accessibility of the RNA II sequence motif for interaction with Com, which prefers the GA dinucleotide to be available in a flexible, single-stranded form (as indicated by high reactivity of GA dinucleotides at the beginning of each strand in SHAPE probing) (Fig 3C). Better accessibility of GA dinucleotide could also explain why Lima’s RNA duplex seems to be a better substrate for Com binding compared to our RNA II. However, a better understanding of Com-RNA interaction will be possible once the complex structure is experimentally determined.

### Comparison of Com-RNA with the Tat-TAR system

As mentioned earlier by Hattman [15], there is a sequence and structure similarity between Com binding region in Mom RNA and TAR RNA (trans-activating region RNA) hairpin involved in interaction with the regulatory protein Tat (trans-activator of transcription) of HIV (human immunodeficiency virus). We noticed that the 5ʹ-CC(N)_2-3_GAG-3ʹ sequence motif recognized by Com is also present in the hexa-loop structure of TAR RNA hairpin. Additionally, in both RNAs the pining base pair is C-G and they have similar stem length (in case of TAR to its bulge region) [15].

TAR interaction with Tat is critical for efficient HIV transcription, gene expression and pathogenesis [44,45]. The TAR hairpin structure is positioned immediately after the transcription start site and stalls viral transcription by RNA polymerase II (Pol II). By binding to TAR, Tat recruits the host super elongation complex (SEC) to the promoter and restores transcription [46][47]. The SEC complex consists of positive elongation factor b (P-TEFb), composed of CDK9 and Cyclin T1 (CycT1), other transcription factors (ELL2 and ENL/AF9) and scaffold proteins (AFF1 and/or AFF4) [48–50]. The studies of Tat-TAR regulatory system revealed that Tat binds directly to a 3-nucleotide bulge region in the major groove of TAR stem by its arginine-rich motif (ARM) and to the loop region in TAR hairpin by cysteine-rich domain [50,51]. Concurrently, Tat binds to the CycT1 of SEC forming a positively charged TAR-interacting surface composed of a helical Tat-TAR recognition motif (TRM) of CycT1 and the Zn^2+^ coordinating loop of Tat [49,50]. The TAR loop in the crystal structure of the complex determined by Schulze-Gahman et *al*. [50] is stabilized by cross-loop hydrogen bonds between C30 and G34 and additional contacts with G33 (corresponding to residues C2, G6 and N5 in the 8-mer Com-binding motif 5ʹ-CCNNNGAG-3ʹ), whereas the remaining loop base moieties of U31, G32 and A35 (corresponding to residues N3, N4, A7 in the 8-mer Com-binding motif) are projected outward from the loop. The protein complex makes a contact with the G32 and G33 bases directly and make extensive contacts with sugar phosphate backbone, suggesting that TAR recognition by SEC is predominantly based on RNA structure [50]. The importance of the TAR loop structure was also proposed based on the mutations in C30 or G34 resulting in a large reduction in CycT1 binding and the fact that the binding could be rescued by another mutation restoring hydrogen-bonding [52].

It is hard to speculate whether Com-Mom and Tat-TAR systems exhibit any analogy in the mode of RNA binding without experimental determination of Com-Mom complex structure. However, our data and the strong similarity between Com target and TAR RNA suggest that the Com binding is also RNA structure-dependent. Due to the almost identical sequence of both hairpins, the Mom loop structure could be stabilized by a cross-loop C-G hydrogen bond and the A nucleotide of the GA dinucleotide could be flipped out as in the TAR loop. The flexibility in the GA dinucleotide context of the unbound form of Mom will be critical for Com binding.

In the structure of Tat-TAR complex (PDB code 6CYT) [50], the key protein residues interacting with the TAR loop are Tyr26 of Tat and Trp258, Arg251, Arg254, Arg259 of CycT1. Searching for similar amino aides combination in Com models [53], we found a positively charged region with Arg8, Asn13, Lys14, and Arg31 located in the proximity of the four cysteine residues involved in the zinc ion coordination. This region could potentially serve as an RNA binding surface in Com. A remaining open question is whether Com requires other factors binding simultaneously to the Mom regulatory element in order to stimulate Mom translation, in analogy to the Tat-TAR system.

Another aspect of Tat-TAR regulation is the proposed recently Chaperna (RNA that provides chaperone function to proteins) activity of TAR RNA [54]. The Tat protein is intrinsically disordered and its interaction with TAR is cooperative [54]. Tat itself exhibits nucleic acid-chaperoning activities [55], and TAR RNA binding, in turn, prevents the Tat protein from misfolding and degradation [54]. It was proposed that TAR RNA may dictate the folding status of Tat, and therefore its interaction with other factors and successful HIV replication in host cells [54]. Such a cooperation could potentially exist also in the Com-Mom regulatory system. The Com protein has a C-terminal intrinsically disordered region, which is dispensable for RNA binding, but could fold upon RNA binding and participate in regulation by RNA structure unwinding or interaction with other factors. In turn, Com in the free, unbound form could be prone to misfolding and degradation (as we observed for GST-fused Com protein after the GST tag cleavage).

Com-Mom and Tat-TAR systems are evolutionary unrelated. However, both systems are essential for the infection and virus propagation in the respective host cells [15][44,45]. The post transcriptional regulation of Mom enables the momification and provide protection against bacterial restriction nucleases, whereas TAR regulation is necessary for efficient HIV transcription. Both, the Mom regulatory region recognition and TAR recognition are sequence- and structure-dependent. If the high similarity of both RNA hairpins is accompanied by similar modes of protein binding, one could anticipate that the inhibitory molecules which interfere with Tat-TAR binding, could also disrupt Com-Mom system, and vice versa. It has been previously shown that methylated oligoribonucleotides which are complementary to the TAR stem-loop [56] or LNA/2′-O-methyl nucleoside analogue aptamers complementary to the loop of TAR can block Tat binding and inhibit the TAR-dependent transcription [57]. In line with these results, we demonstrated that the double-stranded self-complementary version of the Com RNA target is not sufficient for effective Com binding. Whether it could have anti-viral implications needs to be assessed with further biochemical and structural studies.

## Supporting information

S1 Fig Packing of the RNA II duplex in the crystal. Unit cell boundary is shown in black with axes marked. Some nucleotides are labeled for indication of the orientation. Figure prepared in PyMol [43].
(TIFF)

S2 Fig Selected bonds and angles measurement for the RNA II duplex. Non-canonical base pairs are represented in red spheres with the exception of the G•G pair represented in green. (A) Intra-strand hydrogen bonding and base step distances. (B) Inter-strand C1α-C1α distances, and N-C1α-C1α and C1α-C1α-N angles (see text for details). Two numbers are shown when double conformation is present in the structure.
(TIFF)

S1 File Selex sequencing results. (TXT)

S2 File SHAPE reactivity report. (XLSX)

S3 File PDB validation report. (PDF)

## References

[1] Glisovic T, Bachorik JL, Yong J, Dreyfuss G. RNA-binding proteins and post-transcriptional gene regulation. FEBS Lett. 2008;582(14):1977–86. 10.1016/j.febslet.2008.03.004 18342629 PMC2858862

[2] Font J, Mackay JP. Beyond DNA: zinc finger domains as RNA-binding modules. Methods Mol Biol. 2010;649:479–91. 10.1007/978-1-60761-753-2_29 20680853

[3] Hall TMT. Multiple modes of RNA recognition by zinc finger proteins. Curr Opin Struct Biol. 2005;15(3):367–73. 10.1016/j.sbi.2005.04.004 15963892

[4] Brown RS. Zinc finger proteins: Getting a grip on RNA. Curr Opin Struct Biol. 2005;15(1 SPEC. ISS.):94–8. 10.1016/j.sbi.2005.01.006 15718139

[5] Miller J, Mclachlan AD, Klug A. Repetitive zinc-binding domains in the protein transcription factor LiA from Xenopus oocytes. EMBO J. 1985;4(6):1609–14. 4040853 10.1002/j.1460-2075.1985.tb03825.x PMC554390

[6] Omichinski JG1, Pedone PV, Felsenfeld G, Gronenborn AM CG. The solution structure of a specific GAGA factor-DNA complex reveals a modular binding mode. Nat Struct Biol. 1997;4(2):122–32. 9033593 10.1038/nsb0297-122

[7] Isernia C, Bucci E, Leone M, Zaccaro L, Lello D, Digilio G, et al NMR Structure of the Single QALGGH Zinc Finger Domain from the Arabidopsis thaliana SUPERMAN Protein. ChemBioChem. 2003;4:171–80. 10.1002/cbic.200390028 12616630

[8] Stoll R Lee B Debler E Laity J Wilson I et. al Structure of the Wilms tumor suppressor protein zinc finger domain bound to DNA. J Mol Biol. 2007;372(5):1227–45. 10.1016/j.jmb.2007.07.017 17716689

[9] Loughlin FE, Mansfield RE, Vaz PM, McGrath AP, Setiyaputra S, Gamsjaeger R, et al The zinc fingers of the SR-like protein ZRANB2 are single-stranded RNA-binding domains that recognize 5’ splice site-like sequences. Proc Natl Acad Sci U S A. 2009;106(14):5581–6. 10.1073/pnas.0802466106 19304800 PMC2667063

[10] Yang M, Stratford May W, Ito T. JAZ requires the double-stranded RNA-binding zinc finger motifs for nuclear localization. J Biol Chem. 1999;274(39):27399–406. 10488071 10.1074/jbc.274.39.27399

[11] Burge RG, Martinez-yamout MA, Dyson HJ, Wright PE. Structural Characterization of Interactions between the Double- Stranded RNA-Binding Zinc Finger Protein JAZ and Nucleic Acids. Biochemistry. 2014;53:1495–510. 10.1021/bi401675h 24521053 PMC3985865

[12] Lu D, Searles MA, Klug A. Crystal structure of a zinc-finger-RNA complex reveals two modes of molecular recognition. Nature. 2003;426(6962):96–100. 10.1038/nature02088 14603324

[13] Hattman S, Newman L, Murthy HM, Nagaraja V. Com, the phage Mu mom translational activator, is a zinc-binding protein that binds specifically to its cognate mRNA. Proc Natl Acad Sci U S A. 1991;88(11):10027–31. 1835088 10.1073/pnas.88.22.10027 PMC52860

[14] Witkowski RT, Hattman S, Newman L, Clark K, Tierney DL, Penner-Hahn J, et al The zinc coordination site of the bacteriophage Mu translational activator protein, Com. J Mol Biol. 1995;247(4):753–64. 10.1006/jmbi.1995.0178 7723029

[15] Hattman S. Unusual transcriptional and translational regulation of the bacteriophage Mu mom operon. Pharmacol Ther. 1999 12;84(3):367–88. 10665835 10.1016/s0163-7258(99)00042-x

[16] Wulczyn FG, Kahmann R. Translational stimulation: RNA sequence and structure requirements for binding of Com protein. Cell. 1991;65(2):259–69. 1826635 10.1016/0092-8674(91)90160-z

[17] Bellaousov S, Reuter JS, Seetin MG, Mathews DH. RNAstructure: Web servers for RNA secondary structure prediction and analysis. Nucleic Acids Res. 2013 7;41(Web Server issue):W471–4. 10.1093/nar/gkt290 23620284 PMC3692136

[18] Skrisovska L, Bourgeois CF, Stefl R, Grellscheid S-N, Kister L, Wenter P, et al The testis-specific human protein RBMY recognizes RNA through a novel mode of interaction. EMBO Rep. 2007;8(4):372–9. 10.1038/sj.embor.7400910 17318228 PMC1852761

[19] Cavaloc Y, Bourgeois CF, Kister L, Stévenin J. The splicing factors 9G8 and SRp20 transactivate splicing through different and specific enhancers. RNA. 1999 3;5(3):468–83. 10094314 10.1017/s1355838299981967 PMC1369774

[20] Bailey TL, Elkan C. Fitting a mixture model by expectation maximization to discover motifs in biopolymers. Proc Int Conf Intell Syst Mol Biol. 1994 1;2:28–36. 7584402

[21] Frith MC, Saunders NFW, Kobe B, Bailey TL. Discovering sequence motifs with arbitrary insertions and deletions. PLoS Comput Biol. 2008;4(5). 10.1371/journal.pcbi.1000071 PMC2323616 18437229

[22] Lima S, Hildenbrand J, Korostelev A, Hattman S, Li H. Crystal structure of an RNA helix recognized by a zinc-finger protein: an 18-bp duplex at 1.6 A resolution. RNA. 2002;8(7):924–32. 12166647 10.1017/s1355838202028893 PMC1370309

[23] McCaskill JS. The equilibrium partition function and base pair binding probabilities for RNA secondary structure. Biopolymers. 1990;29(6–7):1105–19. 10.1002/bip.360290621 1695107

[24] Tafer H, Höner zu Siederdissen C, Stadler PF, Bernhart SH, Hofacker IL, Lorenz R, et al ViennaRNA Package 2.0. Algorithms Mol Biol. 2011;6:26 10.1186/1748-7188-6-26 22115189 PMC3319429

[25] Wilkinson KA, Merino EJ, Weeks KM. Selective 2 ¢ -hydroxyl acylation analyzed by primer extension (SHAPE): quantitative RNA structure analysis at single nucleotide resolution. Nat Protoc. 2006;1(3):1610–6. 10.1038/nprot.2006.249 17406453

[26] Karabiber F, Mcginnis JL, Favorov O V, Weeks KM. QuShape: Rapid, accurate, and best-practices quantification of nucleic acid probing information, resolved by capillary electrophoresis. RNA. 2013;19(1):63–73. 10.1261/rna.036327.112 23188808 PMC3527727

[27] Low JT, Weeks KM. SHAPE-directed RNA secondary structure prediction. Methods. 2010 10;52(2):150–8. 10.1016/j.ymeth.2010.06.007 20554050 PMC2941709

[28] Darty K, Denise A, Ponty Y. VARNA: Interactive drawing and editing of the RNA secondary structure. Bioinformatics. 2009;25(15):1974–1975. 10.1093/bioinformatics/btp250 19398448 PMC2712331

[29] Xds Kabsch W. Acta Crystallogr Sect D Biol Crystallogr. 2010;66(2):125–32. 20124692 10.1107/S0907444909047337 PMC2815665

[30] Matthews B. W. Solvent Content of Protein. J Mol Biol. 1968;33(August 1967):491–7. 5700707 10.1016/0022-2836(68)90205-2

[31] Kiliszek A, Kierzek R, Krzyzosiak WJ, Rypniewski W. Structural insights into CUG repeats containing the “stretched U-U wobble”: Implications for myotonic dystrophy. Nucleic Acids Res. 2009;37(12):4149–56. 10.1093/nar/gkp350 19433512 PMC2709583

[32] Mccoy AJ, Grosse-kunstleve RW, Adams PD, Winn MD, Storoni LC, Read RJ. research papers Phaser crystallographic software research papers. J Appl Crystallogr. 2007;40:658–74. 10.1107/S0021889807021206 19461840 PMC2483472

[33] Emsley P, Cowtan K. research papers Coot: model-building tools for molecular graphics research papers. Acta Crystallogr Sect D. 2004;D60:2126–32. 10.1107/S0907444904019158 15572765

[34] Murshudov GN, Nicholls RA. research papers REFMAC 5 for the refinement of macromolecular crystal structures research papers. Acta Crystallogr Sect D. 2011;D67:355–67. 10.1107/S0907444911001314 PMC3069751 21460454

[35] Winn MD, Charles C, Cowtan KD, Dodson EJ, Leslie AGW, Mccoy A, et al Overview of the CCP 4 suite and current developments research papers. Acta Crystallogr Sect D. 2011;4449:235–42. 10.1107/S0907444910045749 PMC3069738 21460441

[36] Chen VB, Davis W, Echols N, Headd JJ, Hung L, Kapral GJ, et al PHENIX: a comprehensive Python-based system for macromolecular structure solution Paul D. Adams, Pavel V. Afonine, G ´ PHENIX: a comprehensive Python-based system for macromolecular structure solution. Acta Crystallogr Sect D. 2010;d66:213–21. 10.1107/S0907444909052925 PMC2815670 20124702

[37] Walen T, Chojnowski G, Gierski P, Bujnicki JM. ClaRNA: a classifier of contacts in RNA 3D structures based on a comparative analysis of various classification schemes. Nucleic Acids Res. 2014;42(19). 10.1093/nar/gku765 PMC4231730 25159614

[38] Holbrook S, Cheong C, Tinoco I, Kim S-H. Crystal structure of an RNA double helix incorporating a track of non-Watson-Crick base pairs. Lett to Nat. 1991;353:579–81. 10.1038/353579a0 1922368

[39] Crooks GE, Hon G, Chandonia J, Brenner SE. WebLogo: A Sequence Logo Generator. Genome Res. 2004;14:1188–90. 10.1101/gr.849004 15173120 PMC419797

[40] Chojnowski G, Bujnicki JM, Bochtler M. RIBER / DIBER: a software suite for crystal content analysis in the studies of protein–nucleic acid complexes. 2012;28(6):880–1. 10.1093/bioinformatics/bts003 22238259 PMC3307108

[41] Zheng G, Lu X jun, Olson WK. Web 3DNA—A web server for the analysis, reconstruction, and visualization of three-dimensional nucleic-acid structures. Nucleic Acids Res. 2009;37(SUPPL. 2):240–6. 10.1093/nar/gkp358 PMC2703980 19474339

[42] Rypniewski W, Adamiak DA, Milecki J, Adamiak RW. Noncanonical G(syn)-G(anti) base pairs stabilized by sulphate anions in two X-ray structures of the (GUGGUCUGAUGAGGCC) RNA duplex. Rna. 2008;14(9):1845–51. 10.1261/rna.1164308 18658118 PMC2525959

[43] Schrodinger. The PyMOL Molecular Graphics System, Version 1.3r1. 2010;

[44] Romani B, Engelbrecht S, Glashoff RH. Functions of Tat: the versatile protein of human immunodeficiency virus type 1. J Gen Virol. 2010;91:1–12. 10.1099/vir.0.016303-0 19812265

[45] Clark E, Nava B, Caputi M. Tat is a multifunctional viral protein that modulates cellular gene expression and functions. 2017;8(16):27569–81. 10.18632/oncotarget.15174 28187438 PMC5432358

[46] He N, Liu M, Hsu J, Xue Y, Chou S, Burlingame A, et al HIV-1 Tat and Host AFF4 Recruit Two Transcription Elongation Factors into a Bifunctional Complex for Coordinated Activation of HIV-1 Transcription. Mol Cell. 2011;38(3):428–38. 10.1016/j.molcel.2010.04.013 PMC3085314 20471948

[47] Sobhian B, Laguette N, Yatim A, Nakamura M, Levy Y, Kiernan R, et al HIV-1 Tat Assembles a Multifunctional Transcription Elongation Complex and Stably Associates with the 7SK snRNP. Mol Cell. 2010;38(3):439–51. 10.1016/j.molcel.2010.04.012 20471949 PMC3595998

[48] Schulze-Gahmen U, Lu H, Zhou Q, Alber T. AFF4 binding to Tat-P-TEFb indirectly stimulates TAR recognition of super elongation complexes at the HIV promoter. Elife. 2014;3(e02375):1–13. 10.7554/eLife.02375 PMC4013717 24843025

[49] Schulze-Gahmen U, Echeverria I, Stjepanovic G, Bai Y, Lu H, Schneidman-duhovny D, et al Insights into HIV-1 proviral transcription from integrative structure and dynamics of the Tat:AFF4:P-TEFb:TAR complex. Elife. 2016;5(e15910):1–21. 10.7554/eLife.15910 PMC5072841 27731797

[50] Schulze-Gahmen U, Hurley JH. Structural mechanism for HIV-1 TAR loop recognition by Tat and the super elongation complex. Proc Natl Acad Sci U S A. 2018;115(51):12973–8. 10.1073/pnas.1806438115 30514815 PMC6305006

[51] Berkhout B. Activation of Human Immunodeficiency Virus Type 1 Is Sequence Specific for Both the Single-Stranded Bulge and Loop of the trans-Acting-Responsive Hairpin: a Quantitative Analysis. J Virol. 1989;63(12):5501–4. 2479775 10.1128/jvi.63.12.5501-5504.1989 PMC251225

[52] Richter S, Ping Y, Rana TM. TAR RNA loop: A scaffold for the assembly of a regulatory switch in HIV replication. 2002;99(12). 10.1073/pnas.122119999 PMC122997 12048247

[53] Waterhouse A, Bertoni M, Bienert S, Studer G, Tauriello G, Gumienny R, et al SWISS-MODEL: homology modelling of protein structures and complexes. Nucleic Acids Res. 2018;46(5):296–303. 10.1093/nar/gky427 PMC6030848 29788355

[54] Min JM, Choi HS, Seong BL. The folding competence of HIV-1 Tat mediated by interaction with TAR RNA. RNA Biol. 2017;14(7):926–37. 10.1080/15476286.2017.1311455 28418268 PMC5546542

[55] Kuciak M, Gabus C, Ivanyi-nagy R, Semrad K, Darlix J, Storchak R, et al The HIV-1 transcriptional activator Tat has potent nucleic acid chaperoning activities in vitro. 2008;36(10):3389–400. 10.1093/nar/gkn177 18442994 PMC2425468

[56] Arzumanov A, Walsh AP, Liu X, Rajwanshi VK, Wengel J, Gait MJ, et al Oligonucleotide analogue interference with the HIV-1 Tat protein-TAR RNA interaction. Nucleosides Nucleotides Nucleic Acids. 2001;20(4–7):471–80. 10.1081/NCN-100002321 11563062

[57] Di Primo C, Rudloff I, Reigadas S, Arzumanov AA, Gait MJ, Toulme J. Systematic screening of LNA/2’-O-methyl chimeric derivatives of a TAR RNA aptamer. FEBS Lett. 2007;581:771–4. 10.1016/j.febslet.2007.01.047 17276430

