# Supplementary figures and images for: Specific interaction of zinc finger protein Com with RNA and the crystal structure of a self-complementary RNA duplex recognized by Com
Source: PLoS One. 2019 Apr 25;14(4):e0214481. doi: 10.1371/journal.pone.0214481 (PMC6483171; doi:10.1371/journal.pone.0214481)

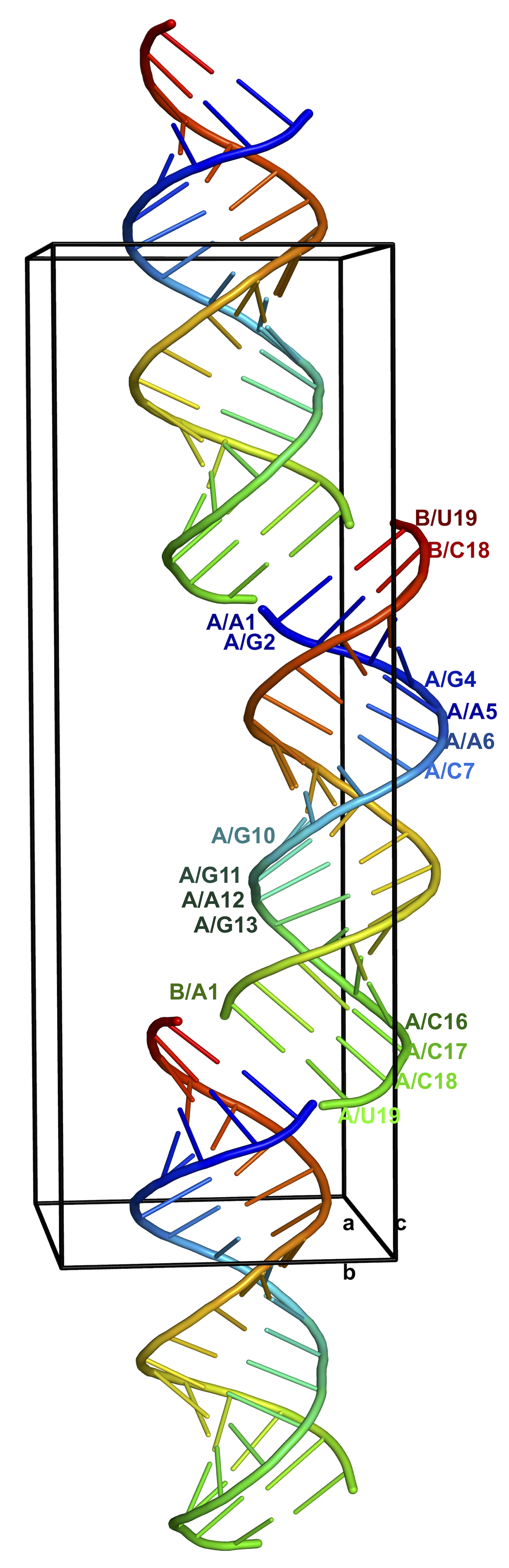

Supplement: S1 Fig — Unit cell boundary is shown in black with axes marked. Some nucleotides are labeled for indication of the orientation. Figure prepared in PyMol [43]. (TIFF) [file pone.0214481.s001.tiff]

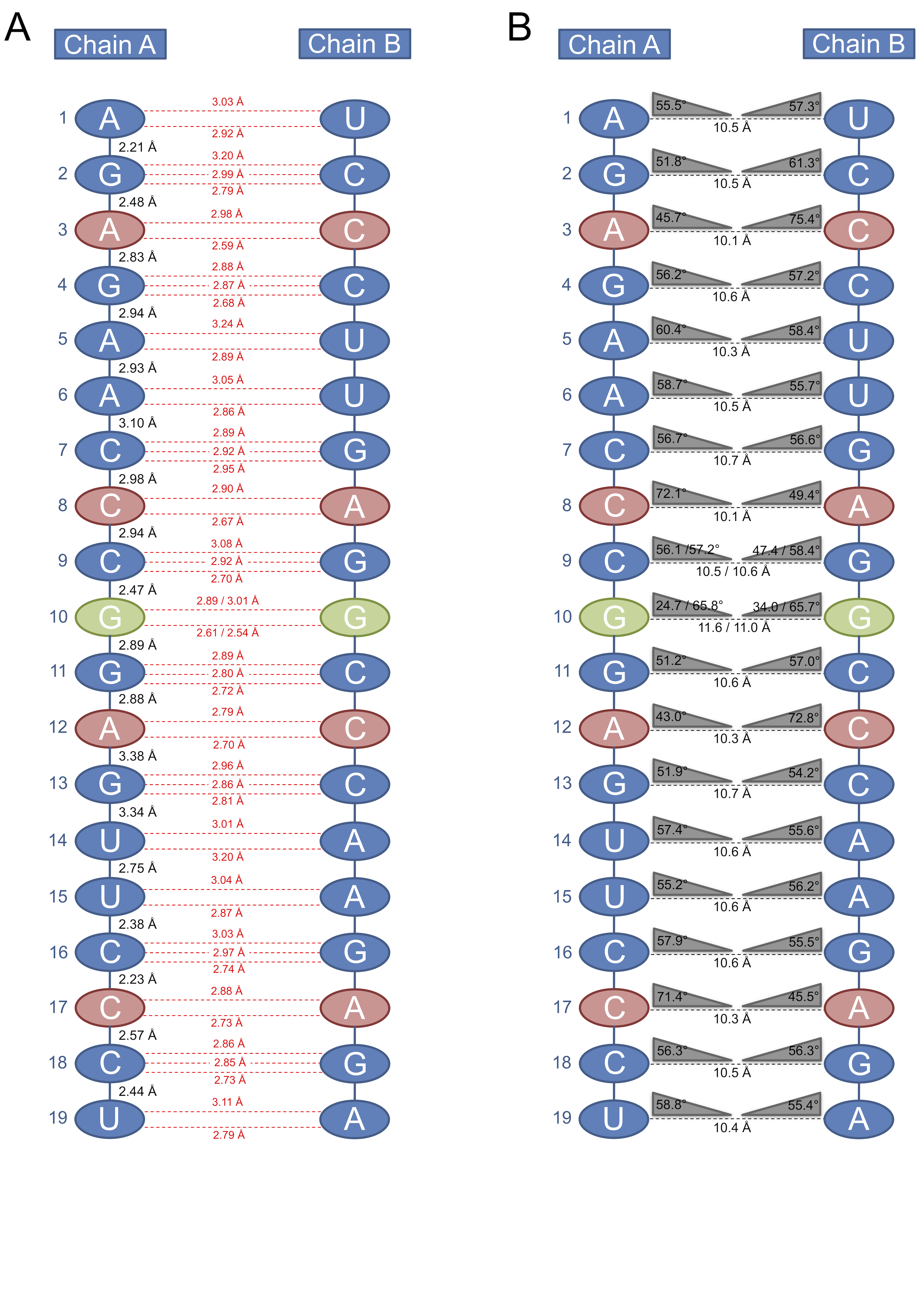

Supplement: S2 Fig — Non-canonical base pairs are represented in red spheres with the exception of the G•G pair represented in green. (A) Intra-strand hydrogen bonding and base step distances. (B) Inter-strand C1α-C1α distances, and N-C1α-C1α and C1α-C1α-N angles (see text for details). Two numbers are shown when double conformation is present in the structure. (TIFF) [file pone.0214481.s002.tiff]
