# Supplementary material for: Specific interaction of zinc finger protein Com with RNA and the crystal structure of a self-complementary RNA duplex recognized by Com
Source: PLoS One. 2019 Apr 25;14(4):e0214481. doi: 10.1371/journal.pone.0214481 (PMC6483171; doi:10.1371/journal.pone.0214481)
Supplement: S3 File — (PDF) [file pone.0214481.s005.pdf]

Full wwPDB X-ray Structure Validation Report (i)

Nov 26, 2018 – 11:54 AM GMT

PDB ID	:	6IA2
Title	:	Crystal structure of a self-complementary RNA duplex recognized by Com
Deposited on	:	2018-11-26
Resolution	:	2.27 Å(reported)

This is a Full wwPDB X-ray Structure Validation Report.

This report is produced by the wwPDB biocuration pipeline after annotation of the structure.

We welcome your comments at *validation@mail.wwpdb.org*
A user guide is available at
https://www.wwpdb.org/validation/2017/XrayValidationReportHelp
with specific help available everywhere you see the (i) symbol.

CONFIDENTIAL VALIDATION REPORT

The following versions of software and data (see references (i)) were used in the production of this report:

MolProbity	:	4.02b-467
Mogul	:	1.7.3 (157068), CSD as539be (2018)
Xtriage (Phenix)	:	1.13
EDS	:	rb-20031633
Percentile statistics	:	20171227.v01 (using entries in the PDB archive December 27th 2017)
Refmac	:	5.8.0158
CCP4	:	7.0 (Gargrove)
Ideal geometry (proteins)	:	Engh & Huber (2001)
Ideal geometry (DNA, RNA)	:	Parkinson et al. (1996)
Validation Pipeline (wwPDB-VP)	:	rb-20031633

1 Overall quality at a glance (i)

The following experimental techniques were used to determine the structure:
X-RAY DIFFRACTION

The reported resolution of this entry is 2.27 Å.

Percentile scores (ranging between 0-100) for global validation metrics of the entry are shown in the following graphic. The table shows the number of entries on which the scores are based.

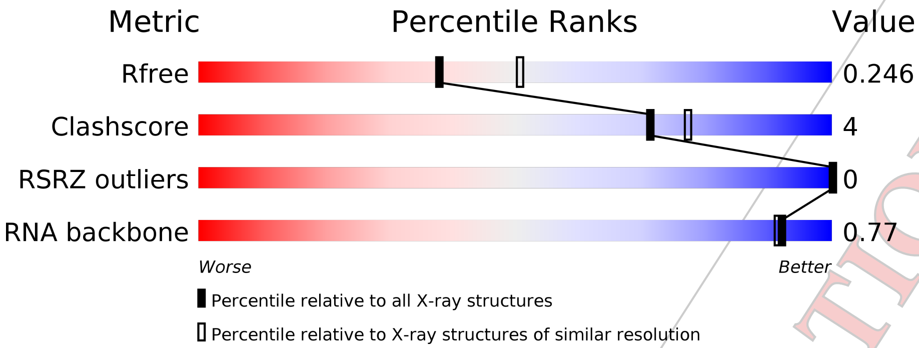

Metric	Whole archive (#Entries)	Similar resolution (#Entries, resolution range(Å))
R_{free}	111664	6121 (2.30-2.26)
Clashscore	122126	6842 (2.30-2.26)
RSRZ outliers	108989	5992 (2.30-2.26)
RNA backbone	2636	1059 (2.76-1.80)

The table below summarises the geometric issues observed across the polymeric chains and their fit to the electron density. The red, orange, yellow and green segments on the lower bar indicate the fraction of residues that contain outliers for >=3, 2, 1 and 0 types of geometric quality criteria. A grey segment represents the fraction of residues that are not modelled. The numeric value for each fraction is indicated below the corresponding segment, with a dot representing fractions <=5% The upper red bar (where present) indicates the fraction of residues that have poor fit to the electron density. The numeric value is given above the bar.

Mol	Chain	Length	Quality of chain
1	A	19	89% 11%
1	B	19	89% 11%

CONFIDENTIAL VALIDATION REPORT

2 Entry composition (i)

There are 4 unique types of molecules in this entry. The entry contains 890 atoms, of which 0 are hydrogens and 0 are deuteriums.

In the tables below, the ZeroOcc column contains the number of atoms modelled with zero occupancy, the AltConf column contains the number of residues with at least one atom in alternate conformation and the Trace column contains the number of residues modelled with at most 2 atoms.

- Molecule 1 is a RNA chain called RNA (5'-R(*AP*GP*AP*GP*AP*AP*CP*CP*CP*GP*GP*AP*GP*UP*UP*CP*CP*CP*U)-3').

Mol	Chain	Residues	Atoms					ZeroOcc	AltConf	Trace
			Total	C	N	O	P			
1	A	19	426	191	79	137	19	0	2	0
1	B	19	448	201	84	143	20	0	2	0

- Molecule 2 is CHLORIDE ION (three-letter code: CL) (formula: Cl).

Mol	Chain	Residues	Atoms		ZeroOcc	AltConf
			Total	Cl		
2	B	1	1	1	0	0

- Molecule 3 is SULFATE ION (three-letter code: SO4) (formula: O_4S).

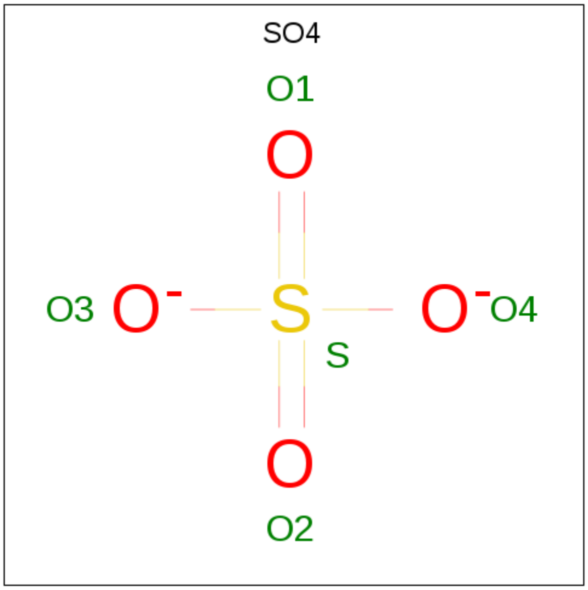

CONFIDENTIAL VALIDATION REPORT

Mol	Chain	Residues	Atoms			ZeroOcc	AltConf
3	B	1	Total 5	O 4	S 1	0	0

- Molecule 4 is water.

Mol	Chain	Residues	Atoms		ZeroOcc	AltConf
4	A	5	Total 5	O 5	0	0
4	B	5	Total 5	O 5	0	0

CONFIDENTIAL VALIDATION REPORT

3 Residue-property plots (i)

These plots are drawn for all protein, RNA and DNA chains in the entry. The first graphic for a chain summarises the proportions of the various outlier classes displayed in the second graphic. The second graphic shows the sequence view annotated by issues in geometryand electron density. Residues are color-coded according to the number of geometric quality criteria for which they contain at least one outlier: green = 0, yellow = 1, orange = 2 and red = 3 or more. A red dot above a residue indicates a poor fit to the electron density (RSRZ > 2). Stretches of 2 or more consecutive residues without any outlier are shown as a green connector. Residues present in the sample, but not in the model, are shown in grey.

• Molecule 1: RNA (5'-R(*AP*GP*AP*GP*AP*AP*CP*CP*CP*GP*GP*AP*GP*UP*UP*CP *CP*CP*U)-3')

Chain A:

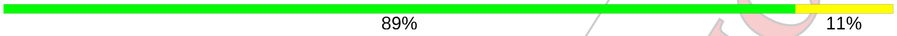

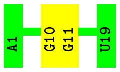

• Molecule 1: RNA (5'-R(*AP*GP*AP*GP*AP*AP*CP*CP*CP*GP*GP*AP*GP*UP*UP*CP *CP*CP*U)-3')

Chain B:

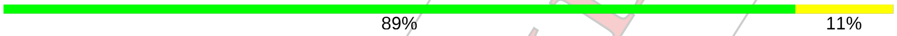

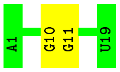

CONFIDENTIAL VALIDATION REPORT

4 Data and refinement statistics (i)

Property	Value	Source
Space group	P 21 21 21	Depositor
Cell constants<br>a, b, c, α, β, γ	31.80Å 38.76Å 101.90Å<br>90.00° 90.00° 90.00°	Depositor
Resolution (Å)	25.55 – 2.27<br>30.85 – 2.27	Depositor<br>EDS
% Data completeness<br>(in resolution range)	99.2 (25.55-2.27)<br>99.4 (30.85-2.27)	Depositor<br>EDS
R_{merge}	0.05	Depositor
R_{sym}	(Not available)	Depositor
$< I/\sigma(I) >$ [1]	2.18 (at 2.26Å)	Xtriage
Refinement program	PHENIX 1.12_2829	Depositor
R, R_{free}	0.212 , 0.246<br>0.212 , 0.246	Depositor<br>DCC
R_{free} test set	310 reflections (5.00%)	wwPDB-VP
Wilson B-factor (Å^2)	46.2	Xtriage
Anisotropy	0.256	Xtriage
Bulk solvent k_{sol}(e/Å^3), B_{sol}(Å^2)	0.28 , 31.3	EDS
L-test for twinning[2]	$< \|L\| >$ = 0.48, $< L^2 >$ = 0.32	Xtriage
Estimated twinning fraction	No twinning to report.	Xtriage
F_o,F_c correlation	0.95	EDS
Total number of atoms	890	wwPDB-VP
Average B, all atoms (Å^2)	41.0	wwPDB-VP

Xtriage's analysis on translational NCS is as follows: *The largest off-origin peak in the Patterson function is 10.32% of the height of the origin peak. No significant pseudotranslation is detected.*

[1] Intensities estimated from amplitudes.
[2] Theoretical values of $< |L| >$, $< L^2 >$ for acentric reflections are 0.5, 0.333 respectively for untwinned datasets, and 0.375, 0.2 for perfectly twinned datasets.

CONFIDENTIAL VALIDATION REPORT

5 Model quality (i)

5.1 Standard geometry (i)

Bond lengths and bond angles in the following residue types are not validated in this section: SO4, CL

The Z score for a bond length (or angle) is the number of standard deviations the observed value is removed from the expected value. A bond length (or angle) with $|Z| > 5$ is considered an outlier worth inspection. RMSZ is the root-mean-square of all Z scores of the bond lengths (or angles).

Mol	Chain	Bond lengths		Bond angles	
		RMSZ	$\#\|Z\| > 5$	RMSZ	$\#\|Z\| > 5$
1	A	0.49	0/500	0.91	0/778
1	B	0.48	0/501	0.92	0/780
All	All	0.49	0/1001	0.92	0/1558

There are no bond length outliers.

There are no bond angle outliers.

There are no chirality outliers.

There are no planarity outliers.

5.2 Too-close contacts (i)

In the following table, the Non-H and H(model) columns list the number of non-hydrogen atoms and hydrogen atoms in the chain respectively. The H(added) column lists the number of hydrogen atoms added and optimized by MolProbity. The Clashes column lists the number of clashes within the asymmetric unit, whereas Symm-Clashes lists symmetry related clashes.

Mol	Chain	Non-H	H(model)	H(added)	Clashes	Symm-Clashes
1	A	426	0	208	1	0
1	B	448	0	228	3	0
2	B	1	0	0	0	0
3	B	5	0	0	0	0
4	A	5	0	0	0	0
4	B	5	0	0	1	0
All	All	890	0	436	4	0

The all-atom clashscore is defined as the number of clashes found per 1000 atoms (including hydrogen atoms). The all-atom clashscore for this structure is 4.

All (4) close contacts within the same asymmetric unit are listed below, sorted by their clash

CONFIDENTIAL VALIDATION REPORT

magnitude.

Atom-1	Atom-2	Interatomic distance (Å)	Clash overlap (Å)
1:B:10[A]:G:H2'	1:B:11[A]:G:H8	1.78	0.49
1:B:10[B]:G:N3	1:B:10[B]:G:H5"	2.29	0.48
1:B:11[B]:G:OP1	4:B:201:HOH:O	2.22	0.41
1:A:10[A]:G:H5"	1:A:10[A]:G:N3	2.36	0.40

There are no symmetry-related clashes.

5.3 Torsion angles (i)

5.3.1 Protein backbone (i)

There are no protein molecules in this entry.

5.3.2 Protein sidechains (i)

There are no protein molecules in this entry.

5.3.3 RNA (i)

Mol	Chain	Analysed	Backbone Outliers	Pucker Outliers
1	A	16/19 (84%)	1 (6%)	0
1	B	16/19 (84%)	0	0
All	All	32/38 (84%)	1 (3%)	0

All (1) RNA backbone outliers are listed below:

Mol	Chain	Res	Type
1	A	11	G

There are no RNA pucker outliers to report.

5.4 Non-standard residues in protein, DNA, RNA chains (i)

There are no non-standard protein/DNA/RNA residues in this entry.

CONFIDENTIAL VALIDATION REPORT

5.5 Carbohydrates (i)

There are no carbohydrates in this entry.

5.6 Ligand geometry (i)

Of 2 ligands modelled in this entry, 1 is monoatomic - leaving 1 for Mogul analysis.

In the following table, the Counts columns list the number of bonds (or angles) for which Mogul statistics could be retrieved, the number of bonds (or angles) that are observed in the model and the number of bonds (or angles) that are defined in the Chemical Component Dictionary. The Link column lists molecule types, if any, to which the group is linked. The Z score for a bond length (or angle) is the number of standard deviations the observed value is removed from the expected value. A bond length (or angle) with $|Z| > 2$ is considered an outlier worth inspection. RMSZ is the root-mean-square of all Z scores of the bond lengths (or angles).

Mol	Type	Chain	Res	Link	Bond lengths			Bond angles		
					Counts	RMSZ	$\#\|Z\| > 2$	Counts	RMSZ	$\#\|Z\| > 2$
3	SO4	B	102	-	4,4,4	0.16	0	6,6,6	0.19	0

In the following table, the Chirals column lists the number of chiral outliers, the number of chiral centers analysed, the number of these observed in the model and the number defined in the Chemical Component Dictionary. Similar counts are reported in the Torsion and Rings columns. '-' means no outliers of that kind were identified.

Mol	Type	Chain	Res	Link	Chirals	Torsions	Rings
3	SO4	B	102	-	-	0/0/0/0	0/0/0/0

There are no bond length outliers.

There are no bond angle outliers.

There are no chirality outliers.

There are no torsion outliers.

There are no ring outliers.

No monomer is involved in short contacts.

5.7 Other polymers (i)

There are no such residues in this entry.

CONFIDENTIAL VALIDATION REPORT

5.8 Polymer linkage issues (i)

There are no chain breaks in this entry.

CONFIDENTIAL VALIDATION REPORT

6 Fit of model and data (i)

6.1 Protein, DNA and RNA chains (i)

In the following table, the column labelled '#RSRZ> 2' contains the number (and percentage) of RSRZ outliers, followed by percent RSRZ outliers for the chain as percentile scores relative to all X-ray entries and entries of similar resolution. The OWAB column contains the minimum, median, 95th percentile and maximum values of the occupancy-weighted average B-factor per residue. The column labelled 'Q< 0.9' lists the number of (and percentage) of residues with an average occupancy less than 0.9.

Mol	Chain	Analysed	<RSRZ>	#RSRZ>2			OWAB(Å^2)	Q<0.9
1	A	19/19 (100%)	-0.02	0	100	100	32, 39, 51, 52	0
1	B	19/19 (100%)	-0.05	0	100	100	34, 42, 49, 50	0
All	All	38/38 (100%)	-0.04	0	100	100	32, 41, 50, 52	0

There are no RSRZ outliers to report.

6.2 Non-standard residues in protein, DNA, RNA chains (i)

There are no non-standard protein/DNA/RNA residues in this entry.

6.3 Carbohydrates (i)

There are no carbohydrates in this entry.

6.4 Ligands (i)

In the following table, the Atoms column lists the number of modelled atoms in the group and the number defined in the chemical component dictionary. The B-factors column lists the minimum, median, 95th percentile and maximum values of B factors of atoms in the group. The column labelled 'Q< 0.9' lists the number of atoms with occupancy less than 0.9.

Mol	Type	Chain	Res	Atoms	RSCC	RSR	B-factors(Å^2)	Q<0.9
3	SO4	B	102	5/5	0.85	0.19	60,65,70,74	5
2	CL	B	101	1/1	0.88	0.07	54,54,54,54	0

6.5 Other polymers (i)

There are no such residues in this entry.

CONFIDENTIAL VALIDATION REPORT
